# Transmitted drug resistance to rilpivirine among antiretroviral-naïve patients living with HIV from northern Poland

**DOI:** 10.7448/IAS.17.1.18929

**Published:** 2014-04-17

**Authors:** Miłosz Parczewski, Anna Urbańska, Katarzyna Maciejewska, Magdalena Witak-Jȩdra, Magdalena Leszczyszyn-Pynka

**Affiliations:** Department of Infectious Diseases, Hepatology and Immune Deficiency, Pomeranian Medical University, Szczecin, Poland

**Keywords:** rilpivirine, NNRTI, drug resistance, transmission, HIV-1 non-B variants, sequencing

## Abstract

**Introduction:**

Rilpivirine (RPV) is a second-generation non-nucleoside reverse transcriptase inhibitor (NNRTI) that was recently approved for the treatment of antiretroviral-naïve individuals with HIV-1 viral load of <100,000 copies/ml. As transmission of the drug resistance mutations to this NNRTI may affect treatment outcomes, the frequency of primary, RPV-associated drug resistance mutations was assessed in this study.

**Methods:**

For the study, 244 viral genome sequences from antiretroviral-naïve individuals were obtained by bulk sequencing. RPV-associated mutations were divided into RPV resistance mutations (K101E/P, E138A/G/K/Q/R, V179L, Y181C/I/V, Y188L, H221Y, F227C and M230I/L) according to the International AIDS Society-USA (IAS-USA) mutation list and variants potentially affecting RPV susceptibility (L100I, K101H/T, E138S, V179F/D/G/T, G190A/E/S, F227L and M230V) based on the *in vitro* and *in vivo* data.

**Results:**

IAS-USA RPV drug resistance mutations were found in 5.3% sequences, with E138A and E138G being the most common (3.7 and 0.8%, respectively), followed by K101E (0.4%) and Y181C (0.4%), with no significant differences in the frequency between subtype B and non-B clades. Mutations potentially reducing RPV susceptibility were found in 2.5% of sequences, and they included V179D (1.6%) and G190A (0.8%), with equal distribution among non-B (*n*=2, 2.5%) and subtype B (*n*=4, 2.5%) clades. Clustering of RPV mutations was infrequent.

**Conclusions:**

Prevalence of RPV-associated drug resistance mutations was low in the analysed sample and did not vary across the subtypes. The frequency of variants with potential influence on RPV susceptibility was similar among non-B variants if compared to B clades. Transmitted drug resistance to RPV is uncommon, which makes this a good option for the treatment of ARV-naïve patients; however, genotype resistance testing should remain compulsory before starting an RPV-based regimen.

## Introduction

Non-nucleoside reverse transcriptase inhibitors (NNRTIs) are commonly affected by transmitted, primary drug resistance [1]. In Europe and worldwide, infection with NNRTI drug-resistant mutants has varied significantly over time, with a recent trend toward stabilization [2,3]. Frequency of NNRTI-transmitted drug resistance has been shown to differ across the HIV-1 subtypes [4,5]. As a result, key international and national treatment guidelines require drug resistance testing prior to the introduction of these agents in antiretroviral treatment-naïve patients [6–8].

Rilpivirine (RPV) has recently been approved for the treatment of naïve subjects living with HIV-1 and a baseline viral load <5 log copies/ml, and it is available both as a co-formulation in combination with tenofovir disoproxyl fumarate/emtricitabine and as a separate tablet for administration with a clinician-selected NRTI background regimen. In the large clinical trials ECHO and THRIVE, RPV regimens proved to be associated with lower frequency of central nervous system-related (dizziness, abnormal dreams and somnolence) and skin-related (rash) adverse events, compared to efavirenz [9]. One of the characteristic features of this substance is the distinct drug resistance profile with full activity retained in the presence of the commonly transmitted and efavirenz-selected K103N mutation [10,11]. Major RPV resistance-associated mutations have been included in the International AIDS Society-USA (IAS-USA) guidelines with K101E/P, E138A/G/K/Q/R, V179L, Y181C/I/V, H221Y, F227C and M230I/L, as well as the recently added Y188L, defined as associated with key resistance [12]. Moreover, in numerous *in vivo* and *in vitro* studies, other candidate drug resistance mutations have been identified, including V90I, L100I, K101H/T, V106L, E138S,V179F/D/G/I/T, V189, G190A/E/S, F227L and M230V [13–18]. It must be noted, that analysis of the ECHO and THRIVE clinical data indicated that the presence of V90I, V106I, V179I and V189I was not associated with virological failure; therefore, these mutations are not likely to be associated with RPV resistance *in vivo* [19].

So far, only a few studies published data on the prevalence of the transmission of drug resistance to RPV, which ranged from 2.4 to 4.6% in antiretroviral treatment-naïve patients [15,16].

Of note, in the ECHO and THRIVE studies of the 456 screening failures with genotypic data, 148 (32%) were due to the presence of mutations. These 148 subjects represent 8.2% of the total screened population (148/1796) [19]. To address the gap in the knowledge on the frequency of RPV-associated mutations, we aimed to analyse the frequency of pre-existing RPV-associated mutations in antiretroviral treatment-naïve subjects living with HIV in northern Poland.

## Material and methods

For the study, 244 viral genome sequences obtained from antiretroviral treatment-naïve HIV-1 patients followed up at the Department of Infectious Diseases, Hepatology and Immune Deficiency, Szczecin, Poland, in the years 1996–2013 were analysed. The protocol of the study was approved by the Bioethical Committee of the Pomeranian Medical University, Szczecin, Poland (approval number KB-0012/08/12). HIV-1 partial *pol* genotyping was performed using the Viroseq 2.8 genotyping kit (Abbott Molecular, Abbott Park, IL) according to the manufacturer's protocol. RPV resistance-associated mutations were divided into key resistance mutations (K101E/P, E138A/G/K/Q/R, V179L,Y181C/I/V, Y188L, H221Y, F227C and M230I/L) based on the IAS-USA drug resistance mutations list update 2013 [12] and potential drug resistance mutations (L100I, K101H/T, E138S, V179F/D/G/T, G190A/E/S, F227L and M230V) based on the clinical trial and *in vitro* data with the exclusion of mutations not associated with virological failure [13–19]. Drug resistance interpretation was performed with the Stanford HIV Drug Resistance Database (HIVDB) (http://hivdb.stanford.edu) [20].

For subtyping, protease and partial reverse transcriptase (RT) gap-stripped nucleotide sequences were used. Sequences were aligned with Clustal X2.0.10 (http://www.clustal.org) software [21], and a set of reference sequences included in the HIV sequence compendium 2012 [22]. An HIV-0 sequence was used as an outgroup. A general time-reversible (GTR)+γ+Γ model with empirical nucleotide frequencies was selected with jModeltest 0.1.1 software. Calculated nucleotide frequencies under this model were as follows: freqA=0.4354, freqC=0.1458, freqG=0.1973, freqT=0.2215 and gamma shape parameter=0.87. Bootstrapped (1000 replicates) trees were inferred under this model to perform initial subtyping, and a neighbour-joining (NJ) tree was constructed using MEGA 5 software. To investigate the existence of transmission clusters with RPV-associated mutations, bootstrapped (1000 replicates) maximum likelihood (ML) trees were inferred under the nearest-neighbour interchange sub-tree algorithm and GTR model with the PHYML v.3.0 software online web server [23]. For calculations of the ML branch support, the approximate likelihood ratio test (aLRT) based on a Shimodaira–Hasegawa-like procedure was used [24]. Clusters were assigned with the aRLT >90% for the external taxonomical units. All trees were visualized in Figtree v.1.2.2. For statistics, a chi-square test with EPI6 Statcalc software was used (Department of Mathematics, University of Louisiana–Lafayette, Lafayette, LA, USA).

## Results

Among analysed sequences, subtype B predominated (163 cases, 67.1%), with subtype D being the second most common in the data set (*n*=36, 14.8%). Of the remaining subtypes, C was found in 15 (6.2%) cases, A1 in 9 (3.7%) and G in 2 (0.8%), with one case each (0.4%) of the F and J clades. Circulating recombinant forms CRF01_AE and CRF02_AG were noted in seven cases (2.9%) each, CRF46_BF in two (0.8%) patients and CRF13_cpx in one (0.4%) case. Of the presented group, all sequences were derived from Polish patients of Caucasian origin, with a predominance of male cases (*n*=163, 66.8%) and a median age of 35 (interquartile range: 27–46) years. No information on the duration of the infection was available.

**Figure 1:**
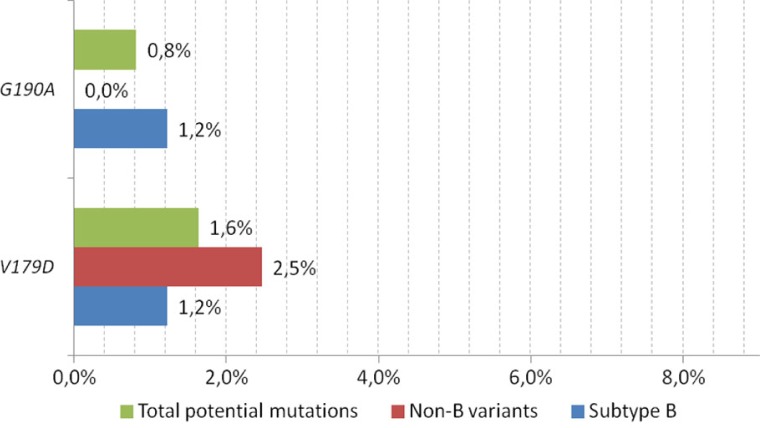
Frequency of rilpivirine drug resistance mutations (IAS-USA 2013 list) among antiretroviral treatment-naïve patients.

Key IAS-USA drug resistance list-defined RPV-associated mutations were found in 13 (5.3%) cases. The most common were the E138A (found in nine cases, or 69.2% of the total key mutations) and E138G (found in two sequences, or 15.5%). Single cases (7.6% each) of K101E and Y181C were noted. The frequency of key mutations was similar between B and non-B variants (4.9 vs. 6.2%, *p*=0.67) (Figure 1). The M184I mutation was not found in the analysed data set. Of note, in the analysed sample, in six patients (2.5%) key NNRTI mutations not associated with RPV were noted, with K103N being the most common (five cases, 2.1%), including one case with a triple K101E/K103N/G190A mutation and one with K103N/P225H/K238T. In one sequence, the Y318F mutation was found.

**Figure 2:**
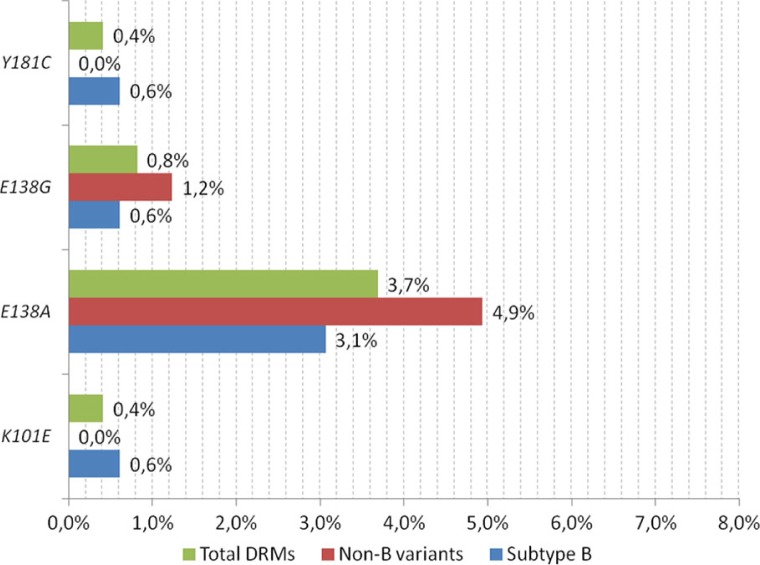
Frequency of variants potentially affecting rilpivirine susceptibility among antiretroviral treatment-naïve patients.

Among variants potentially affecting the RPV susceptibility, the most common were V179I and V179D, which were found in 14 (46.7% of total) and 4 (13.3%) cases, respectively. V90I was noted in 6 (20%) sequences, V189I in 4 (13.3%) and G190A in 2 (6.7%) cases (Figure 2).

Frequency of these mutations was equal among the non-B clades compared to the B clades [2 (2.5%) vs. 4 (2.5%), respectively, odds ratio (OR) 0.99 (95% confidence interval (CI): 0.15–7.99), *p*=n.s]. The total frequency of the RPV drug resistance mutations and variants potentially affecting RPV susceptibility was 7.8%, being similar among the non-B clades (8.6%) compared to the B sequences (7.4%) [OR 1.19 (95% CI: 0.4–3.42), *p*=n.s] (Table 1).

**Table 1:** Frequency of key and potential rilpivirine-associated mutations in the analysed antiretroviral treatment-naïve group across HIV-1 variants

Subtype/recombinant	Key RPV drug resistance mutations, *n* (%)	Potential RPV drug resistance variants, *n* (%)	Any RPV mutation, *n* (%)
**Subtype B**	**8 (4.9)**	**4 (2.5)**	**12 (7.4)**
**Variants non-B (total)**	**5 (6.2)**	**2 (2.5)**	**7 (8.6)**
Subtype A	–	–	–
Subtype C	–	–	–
Subtype D	1 (2.8)	2 (2.5)	3 (8.3)
Subtype G	–	–	–
Subtype J	–	–	–
CRF01_AE	–	–	–
CRF02_AG	4 (57.1)	–	4 (57.1)
CRF13_cpx	–	–	–
CRF46_BF	–	–	–
**Total (B plus non-B)**	**13 (5.3)**	**6 (2.5)**	**19 (7.8)**

In three cases, double RPV-associated mutations with one key mutation and one minor variant were noted (K101E/G190A and V179D/Y181C in subtype B-infected patients and E138G/V179D in a subtype D-infected female). Bolded text shows total B versus non-B subtypes.

According to the Stanford HIVDB, in five (2.1%) cases resistance to RPV was assigned (four cases of intermediate- to low-level resistance and one case of potential low-level resistance).

**Figure 3:**
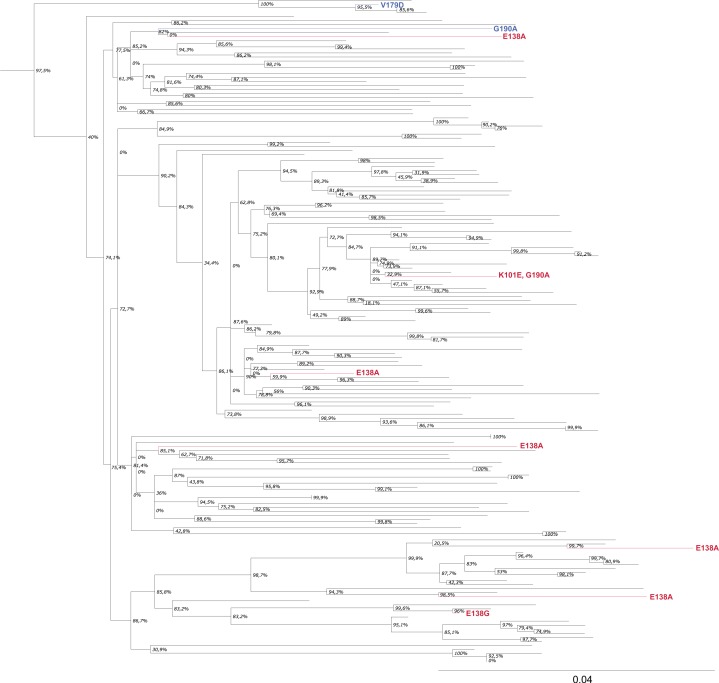
Rilpivirine-associated mutations in subtype B sequences. Taxonomical units with IAS-USA key RPV drug resistance mutations have been coloured in red. Units with mutations potentially affecting RPV susceptibility have been marked in blue.

**Figure 4:**
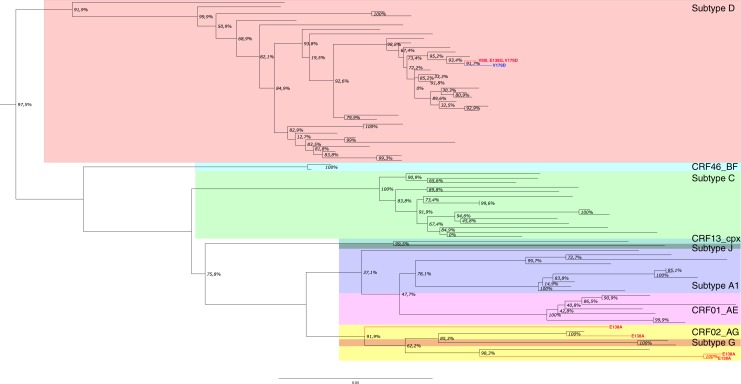
Rilpivirine-associated mutations in non-B clades. Taxonomical units with IAS-USA key RPV drug resistance mutations have been coloured in red. Units with mutations potentially affecting RPV susceptibility have been marked in blue. Each non-B subtype and CRFs have been highlighted with a separate background.

In phylogenetic analysis of the subtype B sequences, no clusters with key drug resistance mutations were noted (Figure 3). Among subtype D-infected patients, one cluster (two sequences) containing E138A and V179D (one with V179D and one E138A+V179D mutant) was found. Among CRF02_AG sequences, a cluster (two sequences) with E138A mutation was observed (Figure 4).

There were no trends over time in the frequency of the RPV-associated mutations, nor were there differences for gender, age or year of infection.

## Discussion

RPV is a first-line choice for the treatment of antiretroviral treatment-naïve patients according to the current guidelines, and it is often selected because of its favourable safety and resistance profile [25]. In this study, frequency of the RPV-associated mutations was analysed in the sample of antiretroviral treatment-naïve patients from northern Poland. This is the first Polish study on RPV resistance and one of the few studies published so far on RPV primary resistance. We also show a high percentage of non-B clades in the analysed data set, which is consistent with previous reports on the prevalence of non-B clades in this region of the country [26].

Frequency of the transmission of NNRI drug resistance has been stable since 2003, being observed in <5% of newly diagnosed patients [1]. It must be noted that the transmission of drug resistance is currently becoming compartmentalized and often occurs in clusters [5,27]. In the data presented, the RPV mutations were rarely transmitted in clusters, with only four clusters with mutations being observed. In two cases, the clusters contained key drug mutations to RPV (one in CRF02_AG and one in subtype D-infected patients).

Observed prevalence of RPV-associated key drug resistance mutations was similar to that found in the studies performed by Lambert-Niclot *et al*. [15]. In our group, E138A was the most common (3%), but the distribution of the IAS-USA-defined drug resistance mutations was not different across the subtypes and recombinants. In a study by Reinheimer *et al*., frequencies of the RPV drug resistance mutations from the German cohort of the Frankfurt Resistance Database were lower – E138K was found in 0.4% of sequences, Y181I/V in 0.9% and K101E in 2.4%. Frequencies of the RPV drug resistance mutations and polymorphic variants were in accordance with the previous data reported not only by Lambert-Niclot *et al*. but also in the pooled ECHO and THRIVE data reported by Vingerhoets *et al*. [15,19]. It should be observed that prevalence of the RPV key drug resistance mutations in our sample is higher than that of the K103N mutation, observed in 2.1% of cases, while etravirine resistance-associated mutations were rare (one sequence each with K238T and Y318F). So far, for Poland the data are very limited, with single cases of NNRTI-transmitted drug resistance [28].

The V179I and V179D variants are not associated with a significant reduction in the RPV fold change susceptibility values. None of the potential RPV mutations observed in our data set alone, if not co-emergent with IAS-USA-listed RPV drug resistance mutations, have been associated with virological failure to RPV [19]. Decreased RPV susceptibility has been observed in *in vitro* study among mutants harbouring Y181C+V179D (a six-fold reduction), V179I+Y181C (3.7-fold), L100I+V179I+Y181C (15.2-fold) and K103N+V179I+Y181C (10.5-fold) [14].

Among double mutants found in our data set, two (K101EK+G190AG and V179D+Y181C) were assigned an intermediate resistance level and one (E138G+V179D) a low level of resistance by the Stanford HIVDB. According to this algorithm, 2% of samples were RPV resistant, which is lower than the previous result of Lambert-Niclot *et al*., but generally in line with the results on the frequency of NNRTI DRM transmission [1].

To conclude, RPV-associated drug resistance mutations associated with virologic failure are infrequent (94.7% of patients with complete RPV susceptibility) among antiretroviral treatment-naïve patients from northern Poland. If all analysed RPV mutations are considered, both potential and IAS-USA defined, the prevalence of resistance-free clades is 92.2%, being similar across HIV subtypes and recombinant clades (92.6% for subtype B versus 91.4% for non-B clades). The clinical significance of potential RPV mutations requires further investigation; however, resistance testing in antiretroviral drug-naïve populations should remain compulsory prior to the initiation of RPV-based treatment, as outlined in the resistance testing and treatment guidelines [29,6].
